# Oligoadenylate synthetase-like aggravated Newcastle disease virus–induced necroptosis in glioma cells

**DOI:** 10.3389/fonc.2025.1574214

**Published:** 2025-04-11

**Authors:** Zecheng Yu, Yuxin Chen, Sisi Chen, Wenjing Ye, Ruirui Li, Yutang Fu, Yangkun Chen, Wenhao Fu, Xianqiao Wei, Qin Yu, Yili Cai, Lingyun Wang, Yuheng Zhang, Huazhong Ying, Fangwei Dai, Wei Han

**Affiliations:** ^1^ School of Medical Imaging, Hangzhou Medical College, Hangzhou, Zhejiang, China; ^2^ Center of Laboratory Animal, Hangzhou Medical College, Hangzhou, Zhejiang, China; ^3^ School of Information Engineering, Hangzhou Medical College, Hangzhou, Zhejiang, China; ^4^ School of Clinical Medicine, Hangzhou Medical College, Hangzhou, Zhejiang, China; ^5^ School of Medical Laboratory and Biological Engineering, Hangzhou Medical College, Hangzhou, Zhejiang, China; ^6^ Engineering Research Center of Novel Vaccine of Zhejiang Province, Hangzhou Medical College, Hangzhou, Zhejiang, China

**Keywords:** NDV, cytolytic activity, glioma, necroptosis, OASL

## Abstract

**Background:**

Newcastle disease virus (NDV) has emerged as a tumor-lysing agent in a variety of cancers. Previous studies have shown that NDV has cytolytic activity in gliomas; however, the underlying mechanisms have not been fully elucidated.

**Methods:**

Comparing the glioma cells LN229 controlled group with the infected group of NDV rLa Sota-GFP strain, we strive to observe the changes in the genome and protein levels as well as the activation of the signalling pathways before and after the infection at the cellular level and at the level of the genes in the transcriptome, to study the molecular mechanism of necroptosis of the NDV-infected lethal LN229.

**Results:**

We found that NDV infection which inhibited glioma cells LN229 proliferation and promoted apoptosis in a dose-dependent manner involved mitochondrial disruption by a molecular mechanism, whereas the Fe^2+^ assay didn’t change. Additionally, the necroptosis inhibitor Nec-1 alleviated the cellular damage caused by NDV during infection of LN229 cells. Using RNA-seq analysis, the necroptosis pathway was significantly enriched in NDV-infected LN229 cells, and the antiviral gene *OASL* (Oligoadenylate synthetase-like) was significantly up-regulated in the apoptotic signalling pathway, which could be directly induced by NDV infection. Knockdown of OASL attenuates NDV infection-induced necroptosis in LN229 cells.

**Conclusion:**

Our study demonstrates that NDV has cytolytic activity on glioma cells by inducing necroptosis. Additionally, targeting upregulation of OASL may provide a novel strategy to enhance necrotic apoptosis in glioma cells after NDV infection.

## 1 Introduction

Oncolytic viruses are naturally occurring or genetically engineered with the capacity to replicate in cancer cells and exert cytotoxic effects (1). Ongoing extensive research has proven that oncolytic viruses selectively kill cancer cells but not normal cells. Such strategy which is currently recognized as a promising biological anticancer therapeutic approach was due to its tumor-specificity, safety, and efficacy. Currently, the use of several viruses, such as adenovirus, coxsackievirus, herpes simplex virus, measles virus, reovirus, and vaccinia virus, has been extensively investigated in various types of advanced cancers and is undergoing clinical trials (2). In the late 20th century, NDV (Newcastle disease virus) emerged as an important research area because of their economic importance and oncolytic potential (3). During the last decade, the oncolytic potential of NDV has been investigated in various animal and human models (4). Since then, NDV has been considered an oncolytic agent for several cancers, including prostate cancer (5), fibrosarcoma (6), cervical cancer (7), breast cancer (8), anaplastic thyroid cancer (9), and lung cancer (10).

Gliomas are among the most prevalent brain tumors that originate from neuroglial progenitor cells. Current therapies, including conventional surgery, chemotherapy, and radiotherapy which are based on nonspecific targeting of proliferating cells, have achieved limited prognosis for patients with glioma (11). Additionally, owing to the unique structure of the blood-brain barrier in the central nervous system (CNS), the majority of anti-tumor drugs are limited to access the brain tissues, which remains the major reason for the lack of progress in the treatment of glioma. Nevertheless, the last several decades of research into oncolytic virus therapy have accelerated the development of new therapies for glioma. Recent advances have suggested the oncolytic potential of several oncolytic viruses for the treatment of gliomas, including NDV (12). Based on the results of preclinical *in vivo* and *in vitro* studies, as well as human clinical trials, NDV has potential tumorolytic activity against gliomas due to its anti-tumor and immunostimulatory properties, which provides a promising avenue for glioma treatment (13). NDV-induced immunogenic cell death (ICD) activates antitumor immune responses and is intimately associated with necroptosis (14). The mechanism by which necrotic apoptosis mediates NDV-induced glioma cells is still unclear, and Necrostatin-1 (Nec-1), which can block necroptosis and target to reduce the expression and phosphorylation of RIP1, was also used in the present study for validation. It has also been found that core genes in protein interaction networks such as *OASL* and *OAS2* may be potential targets for the treatment of gliomas (15). Meanwhile, *OASL* was the only gene significantly up-regulated at 4 and 24 h postinfection after NDV infection of DF-1 cells, and the knockdown of this gene reduced the host’s antiviral gene expression response, significantly increased the viral load of NDV, and resulted in the decreased expression of genes related to apoptosis regulation (*CASP8* and *CASP9*), so it has great potential for targeting OASL in NDV infection (16).

Therefore, in the present work, we explored the molecular mechanism of necrotic apoptosis induced by NDV in LN229 glioma cells *in vitro*, targeting OASL to mediate the anti-tumor response of NDV, and our findings lay the research foundation for the use of NDV as an oncolytic virus for the individualized treatment of glioma patients.

## 2 Methods and materials

### 2.1 NDV and glioma cells treatment

The attenuated La Sota strain of NDV containing GFP protein (rLa Sota-GFP) was constructed and stored in our laboratory. The infectious virus was propagated in 9-day-old embryonated chicken eggs and stored at -80°C after the detection of viral titers. The human glioblastoma cell line (LN229) was provided by Professor Wu (Zhengzhou People’s Hospital, Zhengzhou, China). LN229 cells were cultured in DMEM containing 10% FBS (10439-024, Gibco, Carlsbad, CA, USA) and 1% antibiotics (15240-096, Invitrogen, Carlsbad, CA, USA). Viral infection was initiated by incubating LN229 cells at the indicated multiplicity of infection (MOI) and maintained at 37°C and 5% CO_2_ for 30 min. The medium was then replaced with fresh NDV-free medium.

### 2.2 EdU assay

The proliferation ability of LN229 cells was detected using an EdU incorporation assay kit (Beyotime Biotechnology, Shanghai, China). At 24 h post-starvation, 5 × 10^5^ cells were incubated with a 10 μM EdU solution for 1 h. The cells were then treated with paraformaldehyde, 0.1% Trition-100, and an EdU chromogenic agent. After incubation with Hoechst33342, cells were imaged using a fluorescence microscope.

### 2.3 Flow cytometry

A double dye Annexin V-FITC (FITC-A)/PI kit (550911, BD Biosciences, San Jose, CA, USA) was used to detect cell apoptosis. LN229 cells were infected with different viral titers (0.1, 1, and 10 MOI) of NDV for 24 h, harvested, and washed with PBS. Then, the cells were resuspended in the binding buffer at a density of 2 × 10^5^ cells, mixed with 5 μL of Annexin V-FITC, and incubated for 15 min at room temperature in the dark. The cells were stained with 5 μL PI (μg/mL) for 30 min in the dark. After incubation, the cells were analyzed using a Flow Cytometer (Beckman Coulter, USA) and FlowJo software.

### 2.4 TEM

LN229 cells were fixed in 2% glutaraldehyde for 1 h in the dark at room temperature, followed by storage at 4°C for 48 h. After washing with PBS, the cells were fixed in 1% osmium tetroxide (OsO_4_) for 30 min, dehydrated in a graded series of ethanol, and finally embedded in a TEM copper grid. Cells were visualized using a Hitachi TEM system.

### 2.5 JC-1 staining

JC-1 staining was conducted to assess the mitochondrial membrane potential (MMP) of LN229 cells after infection with NDV. Briefly, after different treatments, LN229 cells were collected and incubated with JC-1 solution (C2003S; Beyotime Biotechnology, Shanghai, China) for 1 h at 37°C. The medium was then removed and replaced with a new medium, followed by detection using a fluorescence microscope (Nikon, TS2R) (17).

### 2.6 Measurement of Fe^2+^ level

Fe^2+^ levels in LN229 cells were measured using an intracellular iron colorimetric assay kit (E1042, APPLYGEN) according to the manufacturer’s instructions. Briefly, LN229 cells were lysed using RIPA lysis buffer and incubated with the Fe^2+^ detection solution provided by the manufacturer. OD_550_ was determined using a microplate reader (Flex Station 3, Molecular Devices, San Jose, CA, USA).

### 2.7 CCK-8 assay

The viability of LN229 cells was measured using the CCK-8 kit (CK04, DOJINDO, Japan) according to the manufacturer’s instructions. Transfected cells were seeded in 96-well plates at a density of 5 × 10^4^ cells/well and infected with or without NDV for 24 h. Next, 10 µL of CCK-8 solution was added to each well, followed by incubation at 37°C for 30 min. The absorbance at 450 nm was measured using a microplate reader.

### 2.8 RNA-seq and differential gene expression analysis

RNA was extracted from NDV-infected LN229 cells (n = 3) versus normal LN229 cells (n = 3) using TRIzol reagent, and the integrity of the total RNA sample was assessed using an Agilent 2100 Bioanalyzer (5067-1511, Agilent Technologies, USA). The library was prepared using the NEBNext^®^ UltraTM RNA Library Prep Kit for Illumina according to the manufacturer’s protocol and its construction was performed at the Novogene Med NGS Clinical Laboratory (Tianjin, China). Subsequently, initially quantified by Qubit 2.0 Fluorometer, it was diluted to 1.5 ng/ul, and then the insert size was detected by Agilent 2100 Bioanalyzer, and after the expectation was met, the effective concentration of the library was precisely quantified (higher than 2 nM) by using qRT-PCR to ensure the quality. Finally, the sequencing was performed using the NovaSeq 6000 (Illumina) at 150 bp paired-end (PE) reads. The sequenced fragments were converted into sequence data (reads) by CASAVA base recognition and filtered for raw data, followed by analysis based on clean data (clean reads). Index of the reference genome was built using Hisat2 (version 2.0.5) which was also used to compare paired-end clean reads to the reference genome. FeatureCounts (version 1.5.0-p3) was used to count the read numbers mapped to each gene. Differential expression analysis between the two comparison combinations was performed using the DESeq2 R package (1.20.0) for samples with biological replicates. The resulting P-values were adjusted using the Benjamini and Hochberg’s approach for controlling the false discovery rate. padj<=0.05 and |log2(foldchange)| >= 1 were set as the threshold for significantly differential expression. All RNAseq expression data have been deposited in Gene Expression Omnibus (GEO) accession number GSE227791.

### 2.9 Functional enrichment analysis

Enrichment analysis is based on the principle of hypergeometric distribution. Gene Ontology (GO) enrichment analysis of differentially expressed genes was implemented by the clusterProfiler R package (3.8.1), in which gene length bias was corrected. GO terms with corrected Pvalue less than 0.05 were considered significantly enriched by differential expressed genes. Meanwhile, the clusterProfiler R package was also used for KEGG pathway enrichment analysis. After correction for multiple comparisons using the Benjamini-Hochberg method, FDR values (false discovery rate, of which padj is a common form) were derived and KEGG pathway enrichment was performed using padj less than 0.05 as the threshold for significant enrichment.

### 2.10 Cell transfection

A specific siRNA targeting OASL (si-OASL: 5’-AAG GAC AGT AAC AAG ACC ACA-3’) was designed and synthesized by Beijing Tsingke Biotech Corporation. The si-OASL or si-scramble were transfected with LN229 cells using Lipofectamine 2000 (12566-014, Invitrogen, Carlsbad, CA, USA). At 12 h post-transfection, the medium was changed and the cells were incubated for another 24 h. The following Q-PCR (Quantitative Polymerase Chain Reaction) experiment was performed to confirm transfection efficiency.

### 2.11 Q-PCR

After transfection, LN229 cells were collected and subjected to total RNA extraction using the TRIzol reagent. A total of 2 μg total RNA was reverse-transcribed into cDNA using a reverse transcription kit (CW0741, CWBIO, China). The reverse transcription product (2 μL) was amplified using primers specific for OASL and β-actin (**Table 1**). The PCR conditions were as follows: initial denaturation at 95°C for 5 min and amplification for 40 cycles: 15 s at 95°C, 15 s at 58°C, and a final extension at 72°C for 30 s at 72°C. The results were calculated using the 2^^-ΔΔCt^ method and analyzed using GraphPad software.

**Table 1:** Sequence of primers used in this study.

Name	Primer sequence
β-actin-F	AGACCTGTACGCCAACACAG
β-actin-R	TTCTGCATCCTGTCGGCAAT
OASL-F	TGAGGCAGGAGCATTTCCAG
OASL-R	CTCCTGAGAACCGTGCCATT

### 2.12 MDA detection

Indicators of oxidative stress in LN229 cells were measured using an MDA assay kit (TBA method, A003-1-2) purchased from Jiancheng (Nanjing, China). Briefly, LN229 cells were cultured in 6-well plates at a density of 2 × 10^5^ cells/well. Following incubation with Tim-AIII for 48h, cells were harvested, resuspended in PBS, and lysed by sonication. The supernatant was collected by centrifugation to detect MDA levels. The absorbance at 532 nm was measured using a microplate reader.

### 2.13 Statistical analysis

A minimum of three biological replicates were performed for each experiment to encompass biological diversity. Statistical analyses were performed using GraphPad Prism 8 (GraphPad Software Inc.). All data are reported as means ± standard deviation (SD). Statistical difference was performed with Student’s t-test (two groups) or one-way ANOVA (three or more groups) followed by Dunnett’s test. Normal distribution was verified using the Shapiro-Wilk test. Statistical *p* was set at p < 0.05. NS means no significant difference, *p < 0.05, **p < 0.01, ***p < 0.001, ****p < 0.0001.

## 3 Results

### 3.1 Infection with NDV inhibits cell proliferation and promotes cell apoptosis in LN229 cells dose-dependently

**Figure 1:**
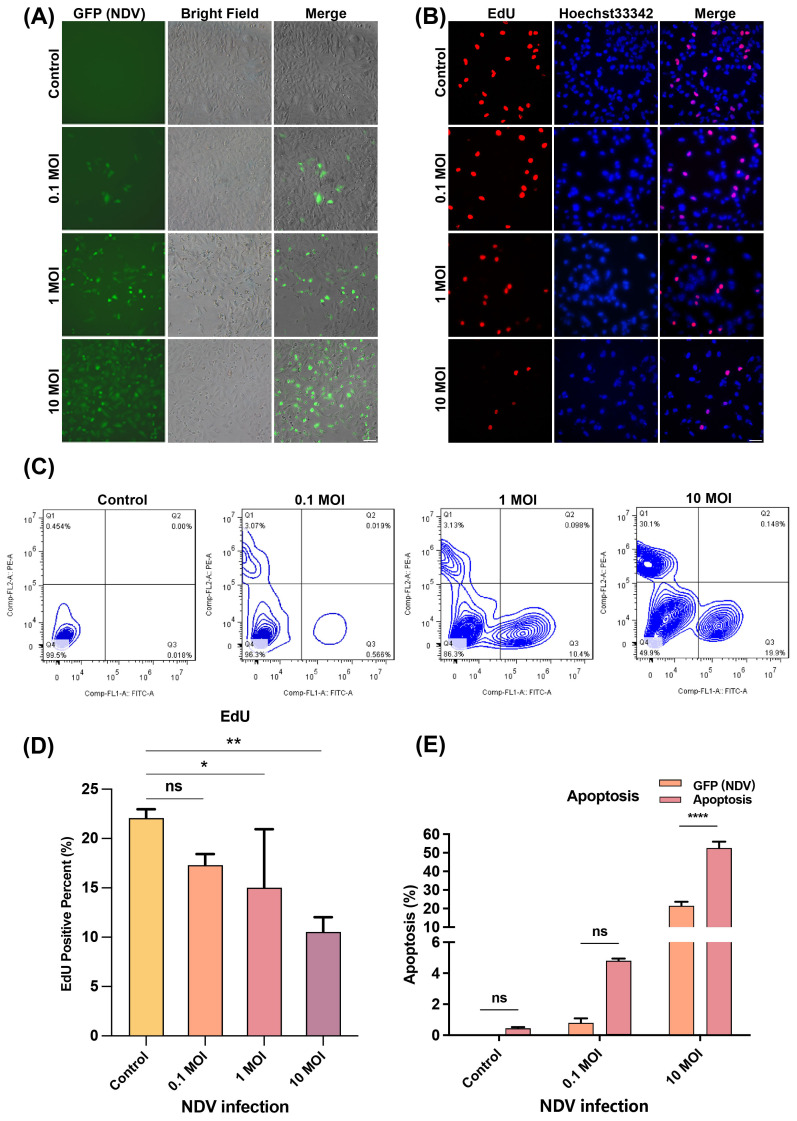
NDV dose-dependently infects and kills LN229 cells. **(A)** Viral infection using recombinant rLa Sota-GFP strain of NDV at different infection multiplicities of 0 MOI, 0.1 MOI, 1 MOI and 10 MOI respectively, revealed that NDV was able to infect LN229 cells. **(B)** EdU-labelled cell proliferation assay at different NDV infection multiplicities showed that NDV infection inhibited the proliferation of LN229 cells by decreasing the proliferating cells with increasing doses of infection. Scale bar=40 μm. **(C)** Flow cytometry was performed on rLa Sota-GFP infected LN229 with an increase in apoptosis of LN229 cells with increasing doses of NDV infection. **(D)** A quantitative count of the cell proliferation assay in panel **(B)** showed that the higher the infection rate of NDV, the lower the proliferation of LN229 cells. **(E)** Quantitative statistics for the flow cytometry detection in panel **(C)** showed that infected cells as well as apoptotic cells increased as the number of infected replicates increased. All data shown represent the means ± SD. n=3. *p < 0.05, **p < 0.01, ****p < 0.0001, ns, no significance.

As shown in **Figure 1A**, as the NDV dose was incremented from 0.1 MOI to 1 MOI, 10 MOI, the rate of LN229 cells being infected increased simultaneously. The EdU staining assay and quantitative statistics showed that NDV infection dose-dependently inhibited the proliferation of LN229 cells (**Figures 1B, D**). In addition, as shown in **Figures 1C** and **E**, it contributed to the induction of apoptosis in LN229 cells with increasing doses of NDV. These results suggest that NDV has oncolytic activity in LN229 cells, infecting and inducing cell apoptosis, and inhibiting proliferation in a dose-dependent manner.

### 3.2 NDV infection promotes necrosis of LN229 cells but with minimal damage to the cell membrane system

**Figure 2:**
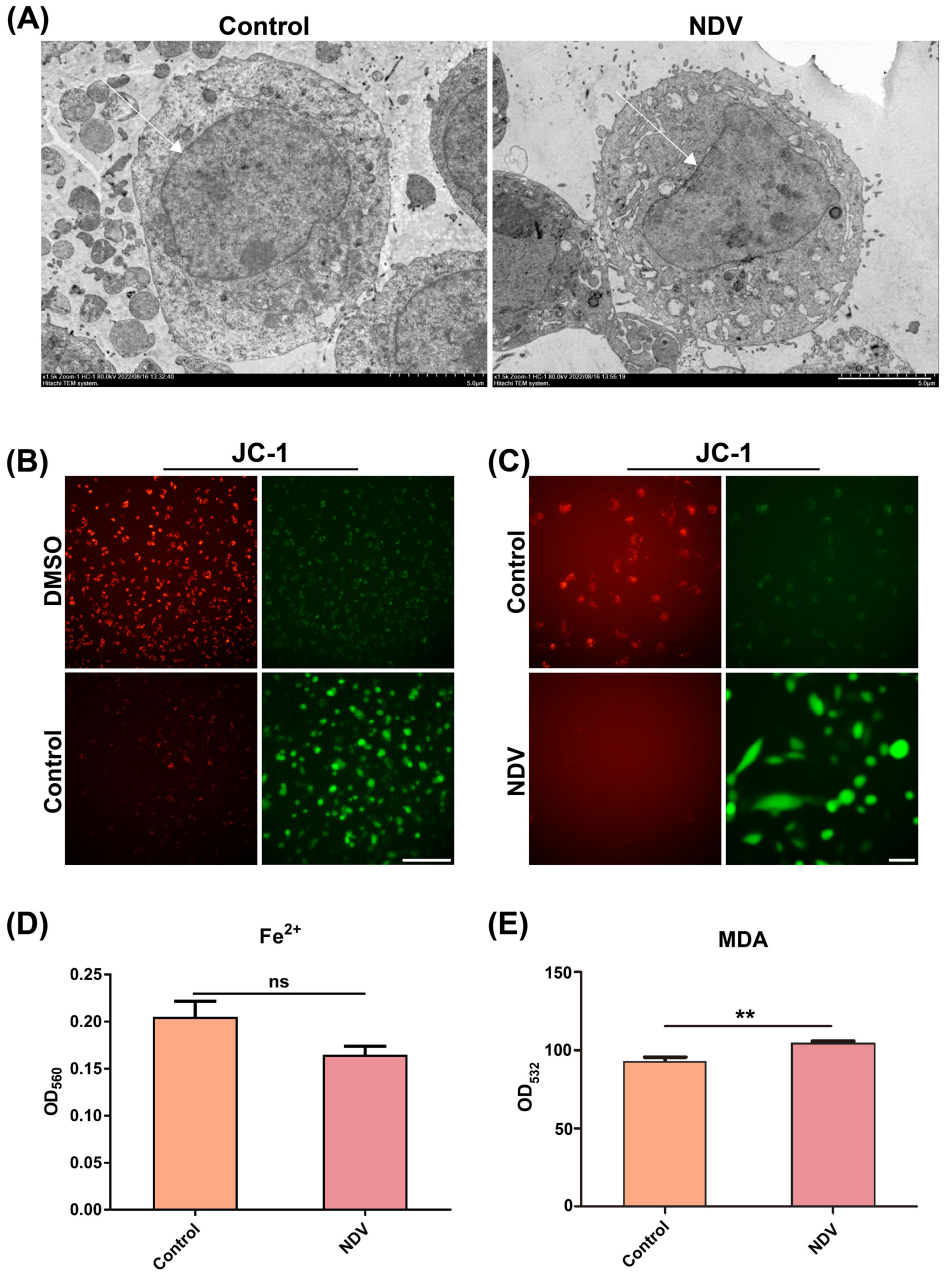
NDV causes necroptosis in LN229 cells. **(A)** The NDV-uninfected control and NDV-infected LN229 cells were examined separately by TEM. Necroptosis is suggested if swelling of organelles, rupture of the cell membrane and breakdown of the cytoplasm and nucleus are observed. Scale bar=5 μm. **(B, C)** Mitochondrial membrane potential of LN229 cells infected with NDV was assessed by JC-1 staining which revealed a significant decrease in red aggregated JC-1 in the mitochondrial matrix and a significant increase in green JC-1 monomers in the cytoplasm after infection with NDV, further indicating that the cells underwent necroptosis. Scale bar=40 μm. **(D)** Measurement of Fe^2+^ levels in cells using a ferric ion colorimetric assay kit revealed no significant iron ion changes in cells after infection with NDV, and no iron death occurred in elevated cells. **(E)** Detection of malondialdehyde MDA, an indicator of oxidative stress with the MDA assay kit revealed that the cells did not undergo significant MDA changes after NDV infection, suggesting that the infection was not achieved by damaging the cell membrane system. All data shown represent the means ± SD. n=3. **p < 0.01, ns, no significance.

As shown by TEM, cytoplasmic organelle damage and cytoplasmic vacuolation were observed in NDV-infected LN229 cells (**Figure 2A**). These findings suggest that NDV causes cell death via necroptosis or ferroptosis. In control LN229 cells, aggregated JC-1 was observed in the mitochondria (red fluorescence). After infection with NDV, increased monomeric JC-1 in the mitochondria (green fluorescence) was found in LN229 cells, implying that NDV infection caused dissipation of ΔΨm and decrease in mitochondrial membrane potential with the beginning of apoptosis (**Figures 2B, C**). These results showed that NDV caused cell death by inducing mitochondrial dysfunction. To exclude ferroptosis, the Fe^2+^ levels were measured. We found that the Fe^2+^ levels did not change after NDV infection (**Figure 2D**). Thus, we speculated that NDV causes cell death by inducing necroptosis. The MDA assay at OD_532_ significantly revealed that LN229 cells did not undergo obvious MDA changes after NDV infection (**Figure 2E**), suggesting that the cell membrane system was not significantly damaged before or after infection (*p* < 0.01).

### 3.3 RIP1 inhibitor Nec-1 blocked the NDV infection-induced LN229 cells necroptosis

**Figure 3:**
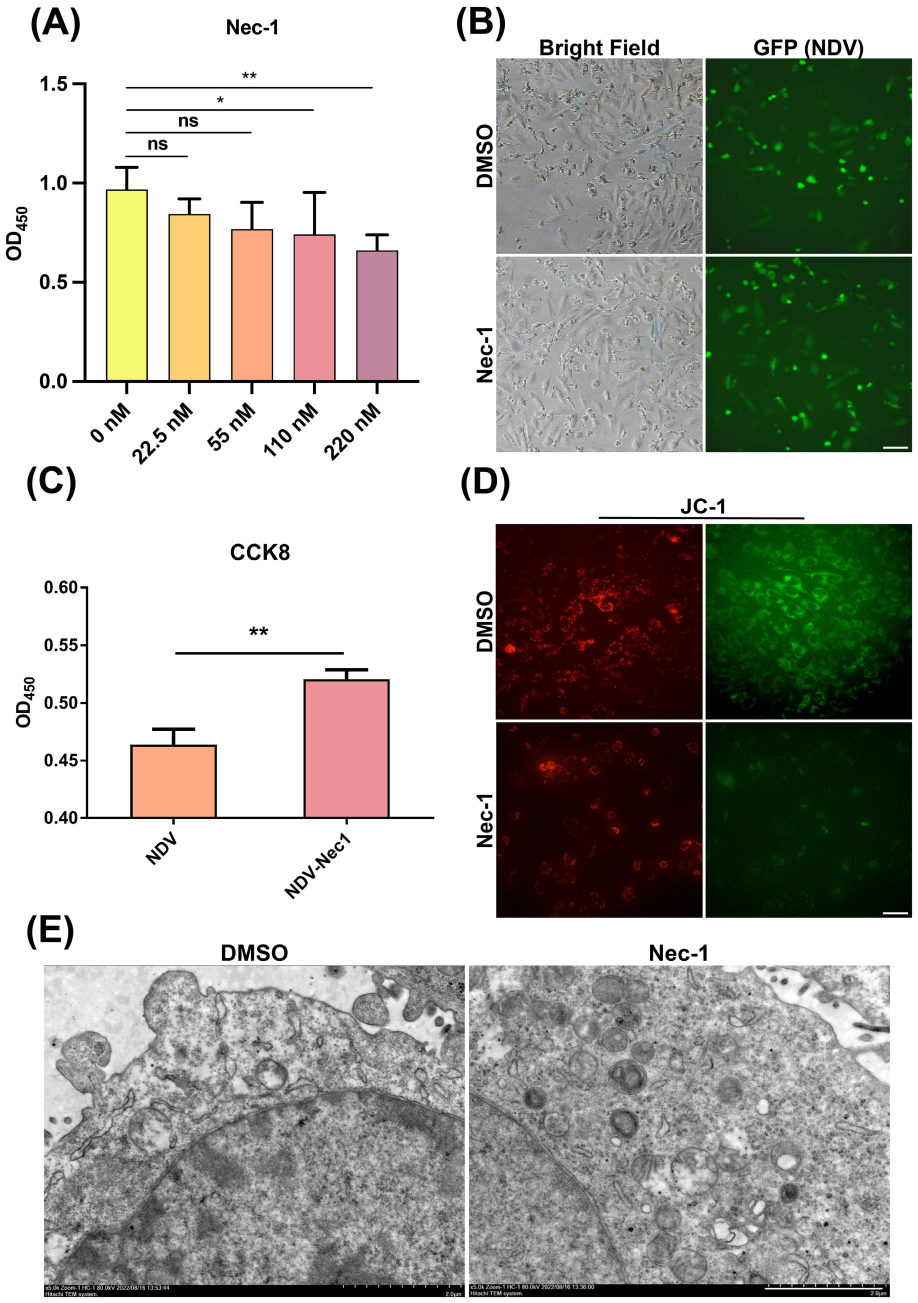
Nec-1, an inhibitor of necroptosis, attenuates NDV-induced damage in LN229 cells. **(A)** Inhibitor toxicity assays with different concentrations of Nec-1 placed at OD_450_ showed no significant toxicity up to 110 nM, so a 22.5 nM concentration of inhibitor will be chosen for later experiments. **(B, D)** The inhibitor of necroptosis, Nec-1, was observed microscopically with a control group stained with DMSO without Nec-1, to compare the fluorescence ratios of the two groups in different colorsit, it was found that the necroptosis inhibitor did not affect NDV infection and the proportion of green fluorescence was similar between the two groups. Scale bar=20 μm. **(C)** CCK-8 kit to determine the cell proliferation viability of NDV-infected group supplemented with Nec-1 showed more proliferating cells in the NDV-infected group with the addition of Nec-1 than in the control group without the added inhibitor of necroptosis. **(E)** Transmission electron microscopy of the cellular structures of the inhibitor-added and control groups in the presence of concurrent NDV infection revealed that cell vacuoles were smaller and mitochondrial damage was relatively weaker in the plus inhibitor group. Scale bar=5 μm. All data shown represent the means ± SD. n=3. *p < 0.05, **p < 0.01, ns, no significance.

To further confirm whether NDV caused cell death by inducing necroptosis, LN229 cells were treated with Nec-1, a necroptosis inhibitor, to block necroptosis in the NDV-infected cells. Nec-1 did not exhibit an obvious cytotoxic effect LN229 cells at concentration of 0-110 nM (**Figure 3A**). In subsequent experiments, NDV-infected LN229 cells were treated with 22.5 nM Nec-1. As shown in **Figure 3B** treatment with 22.5 nM Nec-1 did not affect NDV infection. CCK-8 assay demonstrated that Nec-1 treatment improved the viability of LN229 cells after NDV infection (**Figure 3C**). JC-1 staining assay indicated that NDV-induced mitochondrial dysfunction was attenuated by Nec-1 (**Figure 3D**). Cytoplasmic organelle damage and vacuolation in NDV-infected LN229 cells were diminished after treatment with Nec-1 (**Figure 3E**).

### 3.4 RNA-seq analysis identified the differential expression genes in NDV-infected LN229 cells

**Figure 4:**
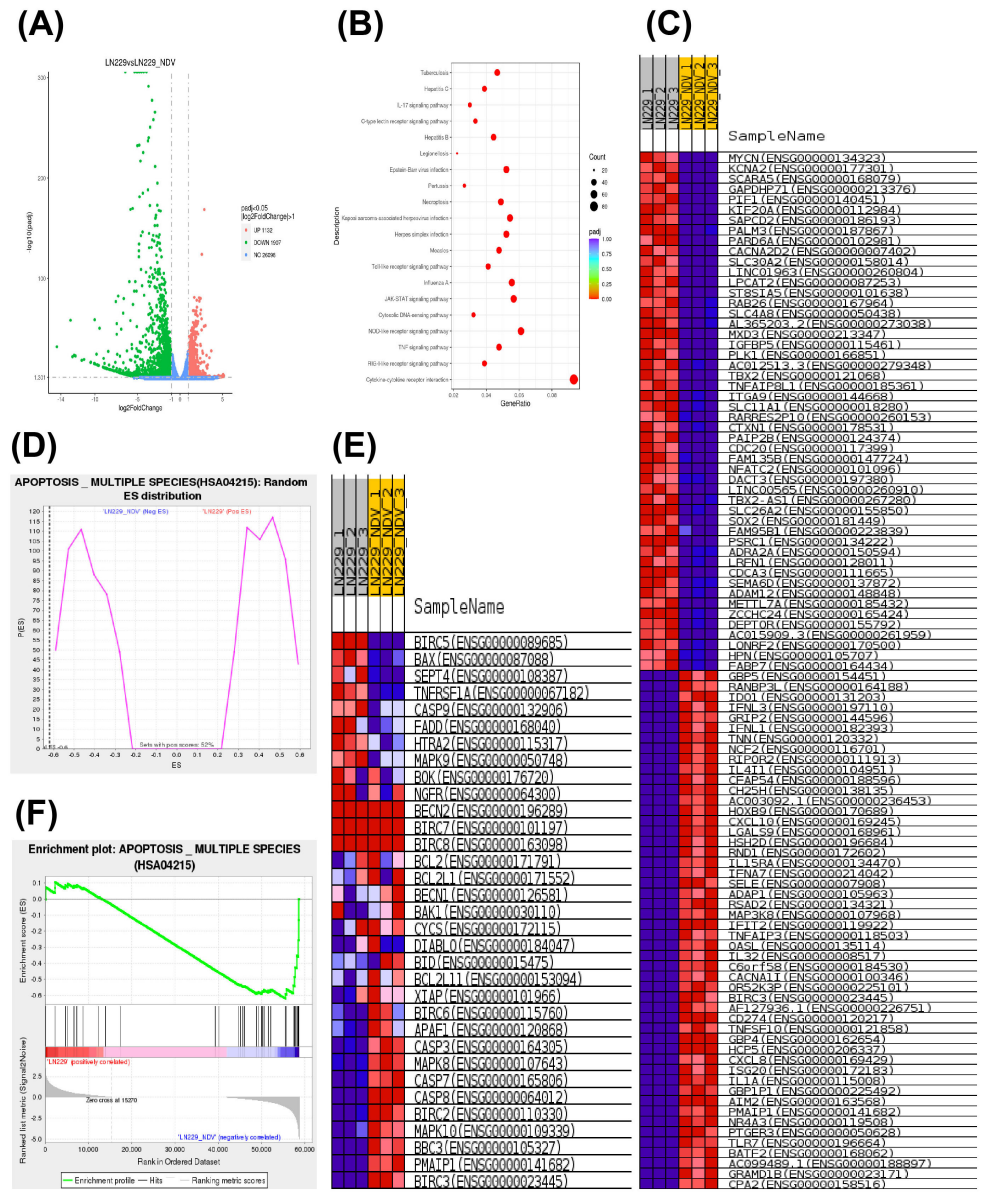
Distribution of associated differentially expressed genes and gene enrichment analysis. **(A)** Volcano plot representing the distribution of DEGs in the GEO database for the LN229 control and NDV-infected LN229 groups (|log2 fold change| >1, *p*<0.05). **(B)** Scatter plot of the 20 most significant KEGG pathways selected based on KEGG enrichment results. **(C, E)** Heat map showing significantly high and low expression genes, including OASL, reflecting both significant enrichment and high expression of genes in apoptotic signalling pathways. **(D, F)** Gene set enrichment analysis (GSEA) of Random ES distribution and Enrichment plot for control and experimental groups in the apoptosis multiple species (HSA04215) pathway.

As shown in the volcano map of differentially expressed genes (DEGs) in NDV-infected LN229 cells, we identified 1132 significantly upregulated genes and 1907 significantly downregulated genes (**Figure 4A**). Next, we performed KEGG and GO analyses of the 3039 DEGs. After selecting the 20 most significant KEGG pathways and plotting scatter plots, the results showed significant enrichment of necroptosis pathways (**Figure 4B**). GO analysis also showed that genes in the apoptotic signalling pathway were significantly upregulated, including *OASL* (**Figures 4C, E**). The expression of the differentially expressed gene *OASL* was significantly up-regulated in the NDV-infected LN229 group, which was approximately 4.98 times higher than that of the control group, while the padj was 0, indicating that the difference was extremely significant (**Supplementary Table 1**). NDV virus-infected LN229 cells recruit OASL signaling pathway to promote necroptosis (**Figures 4D, F**). Therefore, we hypothesized that OASL plays an important role in NDV-infected LN229 cells and is associated with necroptosis.

### 3.5 Knockdown of OASL alleviated NDV infection-induced LN229 cells necroptosis

**Figure 5:**
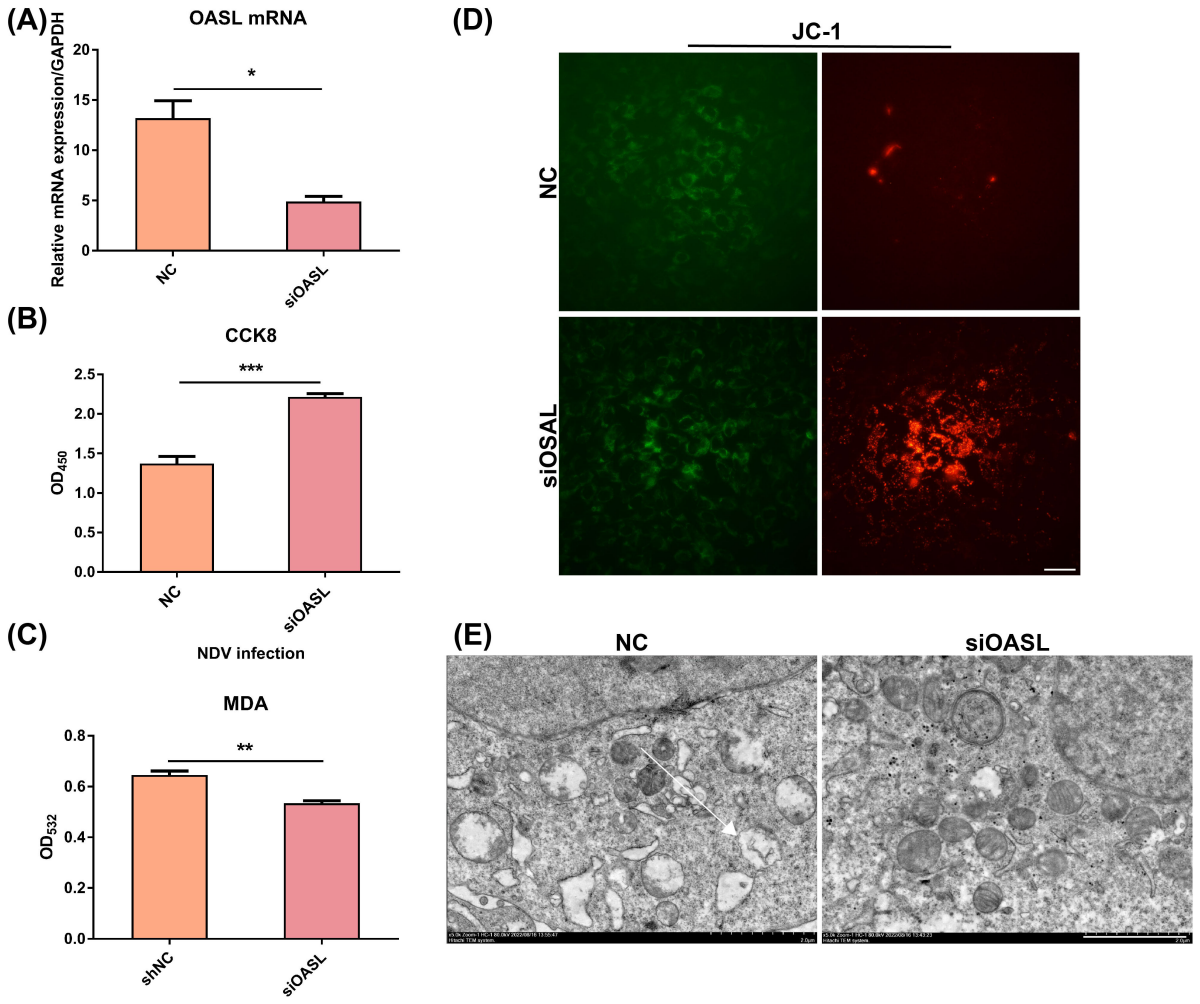
Knockdown of OASL alleviates NDV-induced necroptosis. **(A)** Q-PCR assay verified the success of siRNA knockdown of OASL. **(B)** CCK-8 proliferation assay at OD_450_ compares the number of surviving cells in the knockout OASL group (siOASL group) and the control group (NC group) in the presence of NDV co-infection, and the results showed a higher number of surviving cells in the knockdown OASL group in the presence of NDV co-infection. **(C)** Detection of oxidative products in the knockout OASL group and the control group in the presence of NDV infection at OD_532_ revealed fewer oxidation products in the knockdown OASL group, suggesting less damage to the cell membrane system in this group. **(D)** With the aid of the JC-1 mitochondrial membrane potential assay, the knockdown group was found to have stronger red fluorescence after NDV infection, suggesting a small change in mitochondrial membrane potential. Scale bar=20 μm. **(E)** Transmission electron microscopic observation of cell structure in siOASL group and control group showed that the knockdown group had smaller vacuoles and more intact organelles such as mitochondria after infection with NDV. Scale bar=5 μm. All data shown represent the means ± SD. n=6, *p < 0.05, **p < 0.01, ***p < 0.001.

Next, we constructed OASL-silenced LN229 cells by transfection with si-OASL, and transfection efficiency was confirmed by Q-PCR (**Figure 5A**). Knockdown of OASL significantly (*p* < 0.0001) increased the survival of NDV-infected LN229 cells (**Figure 5B**). The MDA assay demonstrated a reduction in membrane lipid peroxidation products in the knockdown group compared to that in the control group (*p* < 0.01), indicating less damage to the cell membrane system in this group (**Figure 5C**). Aggregated JC-1 (red fluorescence) was significantly increased in the mitochondria of si-OASL-transfected LN229 cells compared to controls, while the intensity of green fluorescence produced by monomeric JC-1 was slightly reduced (**Figure 5D**). Additionally, OASL knockdown attenuated cytoplasmic organelle damage and cytoplasmic vacuolization in NDV-infected LN229 cells (**Figure 5E**). These results suggest that OASL plays a facilitating role in NDV-induced necroptosis of LN229 cells.

## 4 Discussion

Increasing evidence has shown that oncolytic NDV strains may serve as promising therapeutic agents for cancers treatment (4, 18). Yurchenko et al. reported that intratumoral NDV injection in a syngeneic model of mouse Krebs-2 carcinoma reduced tumor progression with increased destructive changes in tumor tissues and volume density of necrotic foci (19). NDV-incorporated with anti-angiogenesis gene VEGF-Trap exerts an enhanced therapeutic efficacy in a colon cancer model, as evidenced by the reduced cell growth ratio and migration ratio in EA.hy926 cells, as well as reduced tumor volume in a colon cancer mouse model (20). Jiang et al. found that the oncolytic NDV inhibits tumor growth in mice bearing THJ-16 T-derived tumors, implying that NDV may have therapeutic potential for anaplastic thyroid cancer (ATC) (9). Lung cell vaccines combined with NDV significantly inhibited tumor growth through an excellent immunotherapeutic effect in lung cancer (21). Importantly, NDV also exerts cytolytic activity against cancers of the nervous system. In a nude mouse model of glioma, recombinant NDV carrying TRAIL had an antitumor effect, as evidenced by a reduction in tumor size and improved behavior in mice (22). The results of these animal experiments suggest that NDV-mediated gene therapy has great potential for the treatment of cancers such as gliomas.

The cytolytic activity of NDV in multiple cancer cells has been investigated in many studies, with the involvement of direct and indirect mechanisms (23). First, the direct mechanisms of NDV-mediated tumor lysis effects include the formation of multinucleated syncytia and activation of diverse signalling pathways, such as the extrinsic and intrinsic apoptotic, ER stress, and MAPK pathways. Second indirect mechanisms underlying NDV-mediated cytotoxicity in cancer cells include the secretion of chemokines and proinflammatory cytokines, which further trigger both innate and adaptive immune responses. For example, targeting lysosomes can exploit the tumor lysis potential of NDV-induced mitochondria-dependent apoptosis (24).

The mechanisms underlying the cytolytic activity of the NDV in gliomas include several aspects. Noemi et al. reported that human glioblastoma (GBM) is susceptible to NDV, which is dependent on CDKN2A-Type I IFN gene cluster co-deletion (12). Meng et al. found that NDV triggered autophagosome formation in U251 glioma cells via the class III PI3K/Beclin-1 pathway, which was then utilized to enhance viral replication during the early stages of infection (25). The oncolytic effects of NDV in glioma cells and glioma stem cells are enhanced by mesenchymal stem cells via the secretion of TRAIL (26).

In the current study, we found that NDV infection in LN229 glioma cells promoted necroptosis, which is a form of programmed cell death. As one of the mechanisms responding to physiological or pathological signals, aberrant activation of necroptosis when cells are exposed to invasion by specific pathogens is thought to be an initiator of a variety of diseases, including cancer (27). Notably, targeting necroptosis is a novel cancer therapy because necroptosis plays an important role in modulating tumorigenesis, metastasis, and tumor progression (28). Thus, modulating the necroptosis of glioma cells might be an important event in the cytolytic activity of NDV in gliomas. We used transcriptome sequencing to mine potential tumor cell apoptosis-inducing target genes and found that OASL was significantly upregulated in glioma cells. Further studies showed that silencing OASL attenuated NDV infection-induced necroptosis in LN229 cells. OASL has recently emerged as an anti-viral gene that exerts various effects on both DNA and RNA viruses (29, 30). OASL is rapidly and directly induced by virus infection and can inhibit cGAS-mediated IFN production, as well as induce RIG-I-mediated IFN induction, thereby controlling antiviral innate immunity and resistance to virus infection (31–33). Unlike mouse Oasl1, human OASL does not bind to IRF7 mRNA, and its deletion decreases RIG-I signaling and enhances the replication of RNA viruses, such as Sendai virus, in human cells, providing a unique antiviral effect (34). However, Chen et al. found that the NDV/HK84 strain significantly inhibited the proliferation and migration of SK-HEP-1 human hepatocellular carcinoma cells through activation of type I interferon signaling, and that many interferon-stimulated genes, including OASL, were markedly up-regulated to participate in anti-tumor immunity, which corresponds to our experimental results in human glioma LN229 cells infected using the rLa Sota-GFP strain of NDV (35). Therefore, we speculated that OASL promotes the tumorolytic effect of NDV and can influence the cytolytic activity of NDV in glioma cells. The experimental results also implied that OASL may be a potential target for enhancing necroptosis induced by NDV infection-induced LN229 cell necroptosis.

The safety of Oncolytic viruses in clinical applications has long been a concern, and numerous studies have shown that NDV is safe and non-pathogenic in humans while rapidly replicating in human cancer cells leading to effective tumor cell lysis, and that its LaSota and Hicher B1 strains, which are highly expressed in chicken embryos, elicit a strong immune response (36). Meanwhile, the LaSota strain of NDV is currently being used worldwide for research and development of vaccine vectors for controlling infectious diseases in animals and humans, so it is representative that rLa Sota-GFP was chosen in this paper. This strain is unaffected by infection of tumor cells at ambient temperature, but is not heat-stable and remains infected for less than 20 min at 56°C so that to construct heat-resistant NDV strains by altering the cleavage site of the F protein or the HN protein, which determines heat stability, would not only be beneficial for storage and transport, but would also reduce the shedding of the virus during infection to provide a better efficacy (37, 38).

In the latest study, based on the discovery that NDV-GT recombinant virus triggers hyperacute rejection, researchers administered NDV-GT intravenously to patients with refractory metastatic cancer, and the results showed that the disease control was up to 90% with durable responses, which proved the feasibility of NDV tumor lysis in treating patients with tumors in the clinic (39). Clinical data have shown that lysogenic NDV can be used to treat some tumors such as gliomas and to stimulate the patient’s immune system. Since the twenty-first century, Steiner et al. treated 23 patients with glioblastoma using ATV-NDV, 91% survived for 1 year, 39% survived for 2 years, and 4% were long-term survivors; Csatary et al. treated four advanced gliomas using the MTH-68/H strain. treated four patients with advanced stage highly gliomas and achieved survival rates of 5-9 years; Freeman et al. treated 11 patients with glioblastoma multiforme using NDV-HUJ intravenously and found that five patients developed grade I/II body heat, which was well tolerated with minimal toxicity (40). However, current therapeutic approaches are still unable to meet the needs of glioma patients with a median survival time of less than 2 years. We found that there was significant expression of *OASL* after NDV infection with LN229, but its therapeutic potential has not yet been explored in depth, and perhaps the use of this target can better utilize the therapeutic effect of NDV on glioma patients in clinic, and improve their survival prognosis. Although NDV has demonstrated a high safety profile in animal and clinical trials and has the potential to target certain tumor cells for killing, but the risks associated with viral mutations, wild-type recombination, and so on should also be emphasized (41). Therefore, in order to improve the safety of clinical application, it is necessary to carry out screening of oncolytic virus strains to enhance its targeting ability and reduce its off-target toxicity and mutation probability. Meanwhile, in the clinical application, since NDV infection can lead to diseases in poultry and birds, it may also bring safety problems to the environment and medical personnel. Consequently, preclinical, translational and clinical applications of NDV not only require a lot of academic research and good technology transfer of the results, but also the related safety tests and regulatory approval.

In conclusion, two aspects of the cytolytic activity of NDV in gliomas were demonstrated in this study. First, NDV infection promoted necroptosis in LN229 glioma cells in a dose-dependent manner, which was blocked by the necroptosis inhibitor Nec-1. Second, NDV infection induces OASL expression, which in turn aggravates NDV infection-induced LN229 cells necroptosis. Taken together, these results indicated that NDV exhibits cytolytic activity in glioma cells by inducing necroptosis. Additionally, targeting OASL may provide a new strategy to enhance the necroptosis of glioma cells after NDV infection.

## Data Availability

The original contributions presented in the study are publicly available. The RNAseq expression data presented in the study are deposited in GEO, accession number GSE227791.
